# Mapping analysis to estimate EQ-5D utility values using the COPD assessment test in Korea

**DOI:** 10.1186/s12955-019-1148-3

**Published:** 2019-06-06

**Authors:** Jaeok Lim, Sang-Eun Choi, Eunmi Bae, Daewon Kang, Eun-A Lim, Gyeong-Seon Shin

**Affiliations:** College of Pharmacy, Korea University, 2511 Sejong-ro, Sejong, 30019 South Korea

**Keywords:** EQ-5D, CAT, COPD, Bootstrap, Korea

## Abstract

**Background:**

There is no research on mapping algorithms between EQ-5D and COPD assessment test (CAT) in Korea. The purpose of this study was to develop mapping algorithms that predict EQ-5D-3 L utility from the CAT in patients with COPD.

**Methods:**

Survey data of 300 COPD patients were collected from three tertiary teaching hospitals in Korea. To predict EQ-5D-3 L utility from the CAT, various models were assessed. Models were developed using randomly split training samples. Subsequently, the models were validated based on root mean square error (RMSE) and mean absolute error (MAE) in validation samples. The models were also validated using the bootstrap method, which involves iterative splitting, training, and validating of the sample data at least 10,000 times. Average RMSEs and MAEs were used as criteria for model selection.

**Results:**

The recommended mapping algorithms were based on ordinary least squares (OLS) regression models, which revealed five CAT items (chest tightness, breathlessness, activity, leaving home, and energy) as statistically significant on the EQ-5D-3 L. The mapping models estimated the overall mean of EQ-5D-3 L utilities effectively, but EQ-5D-3 L utilities for severe (low utility) patients (< 0.6) were overestimated as the observed EQ-5D-3 L utilities were often distributed over 0.6.

**Conclusion:**

Mapping algorithms can be used to predict EQ-5D-3 L utilities from the CAT. However, mapping algorithms should be used cautiously when applied to groups with greater disease severity.

**Electronic supplementary material:**

The online version of this article (10.1186/s12955-019-1148-3) contains supplementary material, which is available to authorized users.

## Introduction

Chronic obstructive pulmonary disease (COPD) has a high global prevalence and mortality, and its socioeconomic burden continues to rise. In 2007 − 2012, the COPD prevalence in Korea was 13.1% in people aged over 40 years. Currently, 20.5% of men and 7.3% of women suffer from COPD [1]. The symptoms of COPD include bronchial closure and shortness of breath, which are known to reduce the mobility of patients, to negatively impact mental health, and, as a consequence, to reduce quality of life (QoL) [2]. In a study comparing the quality of life of patients with chronic diseases, COPD patients scored the lowest [3].

The COPD assessment test (CAT) was designed to provide a simple and reliable measure of health status in COPD patients. The CAT questionnaire consists of eight simple items about coughing, phlegm, chest tightness, breathlessness, home activities, leaving home, sleep problems, and energy. Each item is scored on a scale of 0–5, such that the total CAT score will fall within a range from 0 to 40. A score of 0 represents the best possible health status, and higher scores indicate worsening health. The St. George’s Respiratory Questionnaire (SGRQ) shares several similarities with the CAT, but it is a more exhaustive screening assessment because it contains 50 questions [4]. Recently, the COPD guidelines recommended using the CAT to assess COPD patients. Hence, the use of the CAT is being expanded in many clinical practices and studies [5].

EQ-5D-3 L (developed by the EuroQoL Group) is a measure of health status comprising five dimensions. Each dimension has three levels: no problems (1), some problems (2), and extreme problems (3). EQ-5D-3 L can indicate 243 health status combinations, from 11,111 (perfect health) to 33,333 (worst health). Preference-based utilities can be obtained from the EQ-5D-3 L health status, allowing calculations of the quality-adjusted life year (QALY) for cost–utility analyses. Valuation studies for the 243 EQ-5D-3 L health status indicators were launched in the U in 1995; studies targeting Koreans began in 2006 [6, 7].

The CAT does not show the respondents’ quantitative utilities or preferences because it measures health status related to lung disease. However, measures of quantitative utility are required for economic evaluations, such as cost–utility analysis (CUA). In cases where only CAT data is available for CUA, a mapping algorithm that can estimate quantitative utility measures from the CAT, such as the EQ-5D utility index, can be useful. Previous mapping studies to estimate EQ-5D-3 L utilities for COPD patients have developed algorithms from the SGRQ [8–10], the clinical COPD questionnaire (CCQ) [11], and the CAT [12].

In this report, we describe the first mapping study of Korean patients with COPD. Further, this study used the CAT and EQ-5D-3 L data of 299 patients to develop mapping algorithms. In Korea, an economic evaluation is required to approve new drug reimbursements and guidelines recommend using a Korean-based utility index. Therefore, this mapping algorithm may be useful for economic evaluations of Korean COPD patients and similar patient populations.

## Methods

### Data

A total of 300 COPD patients from three tertiary teaching hospital were surveyed from July–December, 2014. All three hospitals are located in Seoul, the metropolitan city, and therefore most of the patients are urban residents.

The survey was approved by the Institutional Review Board (IRB) of each institute. Patients were eligible for the study if they fulfilled the following criteria: 1) were over the age of 40, 2) had been diagnosed with COPD before 2013, 3) had continuously received outpatient care since January 2013, 4) had an FEV1 (forced expiratory volume in 1 s)/FVC (forced vital capacity) ratio of less than 0.7 immediately after using bronchodilators, and 5) had more than 10 pack-years of smoking history. Patients were excluded if they suffered an acute exacerbation in the six weeks prior to the survey or a cardiovascular event (myocardial infarction or arrhythmias) in the three months prior to the survey.

The survey was conducted by nurses from the respective hospitals using the Korean version of the CAT and EQ-5D-3 L questionnaire. The nurses explained the questionnaires and conducted in-person interviews with the patients. After the interview, the patients’ characteristics (sex, age, duration of COPD, lung function measurement, complication, prescription drugs, resource usage, etc.) were recorded by reviewing their medical records. One of the 300 patients withdrew their consent, so the results from 299 patients were included in this study. For the CAT instrument used in this survey, a Korean version had been evaluated for validity by Lee et al. [13], Hwang et al. [14]. Both evaluations concluded that the Korean version of CAT had good internal consistency and could be used to assess the impacts of COPD on patient health.

Table 1 shows the descriptive statistics of patient characteristics that contains sex, age, BMI, smoking history and FEV1% predicted. The mean age of the patients was 69.2 years, and greater than 74% of the study population consisted of patients over 65 years. The proportion of males was 86.3%. The mean BMI was 22.85, and 8.7% of the patients reported a BMI of less than 18.5 (underweight). Mean smoking history was 36.9 pack-years, and 85% of the patients were current or ex-smokers. The mean duration of COPD was 5.4 years. The FEV1/FVC ratio represents the proportion of a person’s vital capacity that they are able to expire in the first second of forced expiration (FEV1) to the full, forced vital capacity (FVC). The result of this ratio is expressed as FEV1%. FEV1% predicted is defined as FEV1% of the patient divided by the average FEV1% in the population for any person of similar age, sex, and body composition. Less FEV1% predicted value means more severe condition. Severity is divided into four groups based on the FEV1% predicted value. The mean predicted FEV1% was 55.8%, and approximately 90% of patients had moderate or severe COPD (Table 1).

**Table 1:** Patient Characteristics

Variables	n, mean	
Total patients, n	299	
Male, n (%)	258	(86.3)
Female, n (%)	41	(13.7)
Age, mean (sd)	69.16	(8.8)
40–54, n (%)	17	(5.7)
55–64, n (%)	62	(20.7)
65–74, n (%)	132	(44.1)
75-, n (%)	88	(29.4)
Body mass index, mean (sd)	22.85	(3.3)
18.5- (underweight), n (%)	26	(8.7)
18.5–25 (normal), n (%)	198	(66.2)
25–30 (overweight), n (%)	67	(22.4)
30+ (obese), n (%)	8	(2.7)
Smoking history (pack-years), mean (sd)	36.93	(31.3)
current or ex-smoker, n (%)	254	(84.9)
FEV1 (% predicted), mean (sd)	55.85	(17.94)
80+ (mild), n (%)	32	(10.7)
50–80 (moderate), n (%)	156	(52.4)
30–50 (severe), n (%)	90	(30.2)
30- (very severe), n (%)	20	(6.71)

FEV1, forced expiratory volume in 1 s; FVC, forced vital capacity; FEV1%, FEV1/FVC ratio; FEV1% predicted, FEV1% of the patient divided by the average FEV1% in the population

The results of the EQ-5D-3 L and CAT questionnaire survey of 299 people are presented in Table 2. The most frequent response for every EQ-5D-3 L item was 1 (42.1–72.6%), and very few respondents (0.7–2.3%) selected option 3. Eighty-two respondents (27.4%) chose option 1 for all five items. Subsequently, the EQ-5D-3 L utilities were calculated using the method for Korean population developed by Lee et al. [7].

**Table 2:** Summary statistics of EQ-5D utilities and CAT scores

Variable	Mean	SD
EQ-5D utility	0.83	(0.15)
Total CAT score	16.38	(8.96)
Q1: cough	1.68	(1.38)
Q2: phlegm	2.13	(1.42)
Q3: chest tightness	1.59	(1.43)
Q4: breathlessness	3.23	(1.46)
Q5: home activities	1.81	(1.62)
Q6: leaving home	1.82	(1.68)
Q7: sleep	1.74	(1.58)
Q8: energy	2.38	(1.38)

The EQ-5D utility scores were calculated using the equation from Lee et al. [7]. Eight each CAT item is scored on a scale of 0–5, and the total CAT score ranges from 0 to 40

EQ-5D-3 L utility = 1–0.050 - 0.096 M2–0.418 M3–0.046SC2–0.136SC3–0.051UA2-0.208UA3–0.037PD2–0.151PD3–0.043 AD2–0.043 AD3–0.050 N3 (M2, mobility level 2; M3, mobility level 3; SC2, self-care level 2; SC3, self-care level 3; UA2, usual activities level 2; UA3, usual activities level 3; PD2, pain or discomfort level 2; PD3, pain or discomfort level 3; AD2, anxiety or depression level 2; AD3, anxiety or depression level 3; N3, any dimension on level 3)

The mean of utility scores was 0.83 (SD = 0.15). The mean of total CAT scores was 16.38, with scores ranging from 0 to 38. Among the eight items, the fourth item (breathlessness) was calculated as the highest (severe) average score at 3.23, and the lowest average score was reported for the first item (cough) at 1.68. There was no missing value in the variables used for this study.

### Model development

The mapping models were developed using the EQ-5D-3 L utilities as the dependent variables and either the total CAT score or eight scores of each CAT item as the explanatory variables in the following formulas:

$$ \bullet \mathrm{Model}\kern0.28em 1:\mathrm{EQ}-5\mathrm{D}-3\mathrm{L}\kern0.28em \mathrm{Utility}=\mathrm{a}+\mathrm{b}1\ast \mathrm{total}\left(\mathrm{CATscore}\right)+\mathrm{c}1\ast \mathrm{age}+\mathrm{c}2\ast \mathrm{sex} $$

$$ \bullet \mathrm{Model}\kern0.28em 2:\mathrm{EQ}-5\mathrm{D}-3\mathrm{L}\kern0.28em \mathrm{Utility}=\mathrm{a}+\mathrm{b}1\ast \mathrm{total}+\mathrm{b}2\ast {\mathrm{total}}^2+\mathrm{c}1\ast \mathrm{age}+\mathrm{c}2\ast \mathrm{sex} $$

$$ \bullet \mathrm{Model}\kern0.28em 3:\mathrm{EQ}-5\mathrm{D}-3\mathrm{L}\kern0.28em \mathrm{Utility}=\mathrm{a}+\mathrm{b}1\ast \mathrm{Q}1+\mathrm{b}2\ast \mathrm{Q}2+\cdots +\mathrm{b}8\ast \mathrm{Q}8+\mathrm{c}1\ast \mathrm{age}+\mathrm{c}2\ast \mathrm{sex} $$

Models 1 and 2 used the total CAT score, age, and sex as explanatory variables. In contrast, Model 3 used eight scores of each CAT item instead of the total CAT score. Backward stepwise selection of explanatory variables was used with significance defined as α = 0.05. The following estimation methods were used: ordinary least squares (OLS), generalized linear models (GLM), Tobit models, and beta regression. Because EQ-5D-3 L utilities have skewed and censored values, we used and compared GLM, Tobit and beta regression as well as OLS. The probability distributions and link function of GLM that we investigated are Gaussian-log, Poisson-log, gamma-inverse, quasi-identity.

A two-part model was also considered as an alternative estimation method to analyze skewed data [8]. In this study, a large proportion (27%) of observed EQ-5D-3 L utilities had a value of 1, which indicated perfect health status. The first part of the two-part model, logistic regression, would determine the probability of having a perfect health status. The second part would use previous OLS, GLM, Tobit and beta regression estimations to predict EQ-5D-3 L utilities. The EQ-5D-3 L utility score was calculated using the following equation:

$$ \bullet \mathrm{Two}-\mathrm{partmodel}:\mathrm{EQ}-5\mathrm{D}-3\mathrm{L}\kern0.28em \mathrm{Utility}=\mathrm{P}\left(\mathrm{perfecthealth}\right)+\left[1-\mathrm{P}\left(\mathrm{perfecthealth}\right)\right]\ast \mathrm{Predicted}-\mathrm{EQ}-5\mathrm{D}-\mathrm{Utility} $$

P(perfect health) is the probability of perfect health obtained from logistic regression. The Predicted-EQ-5D-3 L-Utility value is derived from previous OLS, GLM, Tobit and beta regression estimations.

All statistical analyses were conducted using R (ver 3.3.3; R Foundation for Statistical Computing).

The R code used for this analysis is provided as Additional file 2.

### Model validation

The datasets of 299 COPD patients were randomly split into a training set of 150 patients (50%) and a validation set of 149 patients (50%). The training dataset was used to develop the models. The validation set was used to validate the models through calculations and comparisons of root mean square errors (RMSE) and mean absolute errors (MAE).

The bootstrap method was used to generate a robust estimate of RMSE and MAE in the limited sample size. The previously described framework, which consists of random splitting, training and validation, was iterated 10,000 times to collect 10,000 RMSEs and MAEs. The means of the collected RMSEs and MAEs were used as criteria for model selection. As such, the model with the lowest RMSE or MAE was selected as the most suitable method.

## Results

### Model development

Table 3 presents the results of mapping models using OLS and Gaussian log-link GLM. All RMSEs and the MAEs values represent the means of 10,000 bootstrap values. Among the four types (Gaussian-log, Poisson-log, gamma-inverse, and quasi-identity) of GLM results, the Gaussian-log model generated the best (lowest RMSE and MAE) values. Both the Tobit and beta regression reported higher RMSE and MAE than OLS or GLM. Additionally, the two-part models showed worse performance (higher RMSE and MAE) than the corresponding single equation models. The results of Tobit and beta regression that are not presented in Table 3 are provided as Additional file 1.

**Table 3:** Comparison of the models based on the means of RMSEs, MAEs

	Single equation		Two-part	
Model	RMSE	MAE	RMSE	MAE
Total CAT score model				
OLS1	0.1112	0.0816	0.1153	0.0830
OLS2	0.1114	0.0836	0.1159	0.0845
GLM1	0.1117	0.0811	0.1154	0.0822
GLM2	0.1115	0.0831	0.1158	0.0840
Selected items model				
OLS3	0.1086	0.0793	0.1121	0.0784
GLM3	0.1092	0.0787	0.1123	0.0776

*MSE* mean squared error, *MAE* mean absolute error, *OLS* ordinary least squares, *GLM* generalized linear model; OLS1 and GLM1 used the total CAT score as the explanatory variable. OLS2 and GLM2 used the total CAT score and the square term of total CAT score as the explanatory variable. OLS3 and GLM3 used the backward stepwise selected CAT questions as explanatory variables. Five selected questions for OLS3 and GLM3 are Q3, Q4, Q5, Q6, Q8

Among the total CAT score models (Models 1 and 2), OLS1 resulted in the lowest RMSE and GLM1 resulted in the lowest MAE. Model 2, which included the square of the total CAT score as an explanatory variable, performed worse than Model 1, which only included the total CAT score. As for the selected items model (Model 3), OLS3 resulted in lower RMSE and GLM3 resulted in lower MAE. Due to the simplicity of use as well as the accuracy of the estimation, we would recommend OLS1 and OLS3 models rather than GLMs. Of which, the selected CAT items model (OLS3) provided more accurate estimates than the total CAT score model (OLS1). However, the OLS1 model must be used to estimate EQ-5D-3 L utilities when the total CAT score is the only known value.

### Recommended models

Table 4 presents the recommended mapping models—OLS1 and OLS3 models. To create a best-fit mapping algorithm for EQ-5D-3 L utility predictions, OLS equations were estimated using the full 299 patient dataset. Both models included the age variable as a negative coefficient value. The sex variable was not statistically significant and was excluded. In the selected items model, the third, fourth, fifth, sixth, and eighth CAT items had significantly negative effects on the EQ-5D-3 L utilities, whereas the other items did not. The significant effects of the third, fifth, sixth and eighth CAT items were consistent with results from a previous mapping study by Hoyle et al. [12]. In this study, one additional item (breathlessness) was included in the model. The equations of the two recommended mapping models are as follows.

$$ {\displaystyle \begin{array}{l}\bullet \mathrm{TotalCATscoremodelOLS}1\\ {}\mathrm{EQ}-5\mathrm{D}-3\mathrm{L}\kern0.28em \mathrm{Utility}=1.1376-0.0103\mathrm{total}\left(\mathrm{CATscore}\right)-0.0020\mathrm{age}\end{array}} $$

**Table 4:** Recommended mapping models

	Estimate	S.E	*P*-value
Total CAT score model OLS1			
Intercept	1.1376	0.0513	0.000
CAT	−0.0103	0.0007	0.000
age	−0.0020	0.0007	0.007
Selected items model OLS3			
Intercept	1.0661	0.0518	0.000
Q3: chest tightness	−0.0097	0.0053	0.067
Q4: breathlessness	−0.0120	0.0059	0.042
Q5: home activities	−0.0168	0.0069	0.015
Q6: leaving home	−0.0255	0.0069	0.000
Q8: energy	−0.0125	0.0057	0.030
age	−0.0011	0.0007	0.141

$$ {\displaystyle \begin{array}{l}\bullet \mathrm{SelecteditemsmodelOLS}3\\ {}\mathrm{EQ}-5\mathrm{D}-3\mathrm{L}\kern0.28em \mathrm{Utility}=1.0661-0.0103\mathrm{Q}3-0.0120\mathrm{Q}4-0.0168\mathrm{Q}5-0.0255\mathrm{Q}6-0.0125\mathrm{Q}8\end{array}} $$

Five items whose responses had significant effects on the EQ-5D-3 L utility scores

**Table:** 

Q3: My chest does not feel tight at all (very tight) Q4: When I walk up a hill or one flight of stairs I am not breathless (very breathless) Q5: I am not limited doing any activities at home (very limited) Q6: I am confident leaving my home despite my lung condition (not at all confident) Q8: I have lots of energy (no energy at all)	

**Fig. 1:**
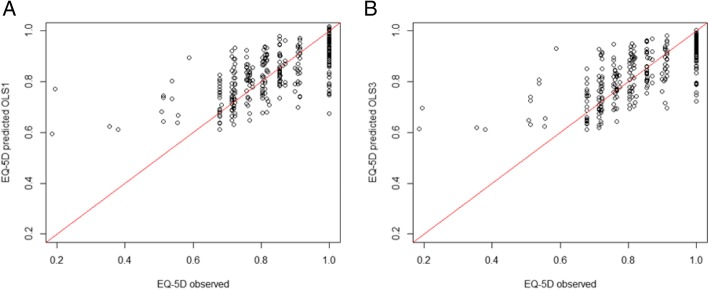
Scatter plots of the observed and predicted EQ-5D utility scores (**a**) Total CAT score model OLS1 (**b**) Selected items model OLS3

Scatter plots of predicted and observed EQ-5D-3 L utilities are shown in Fig. 1. As illustrated by the scatter plots, the mapping model overestimated EQ-5D-3 L utilities for severe status patients (observed EQ-5D-3 L utilities < 0.6). The range of observed EQ-5D-3 L utilities (0.095 − 1) differed from the range of predicted EQ-5D-3 L utilities (0.6 − 1).

### Disease severity

Table 5 presents observed and predicted EQ-5D-3 L utilities categorized by COPD severity. Both OLS1 and OLS3 produced similar utilities to EQ-5D-3 L for moderate and severe health status; however, the utility was underestimated for mild COPD and overestimated for very severe COPD.

**Table 5:** Mean(SD) observed EQ-5D utility compared to mean predicted EQ-5D utility scores by disease severity

	Disease severity			
	mild	moderate	severe	very severe
No. of patients	32	156	90	20
observed	0.911(0.111)	0.860(0.117)	0.792(0.156)	0.665(0.169)
OLS1	0.870(0.082)	0.854(0.089)	0.797(0.094)	0.751(0.084)
OLS3	0.888(0.078)	0.856(0.092)	0.792(0.098)	0.721(0.080)

## Discussion

This study developed mapping algorithms to predict EQ-5D-3 L utility using CAT responses from 299 Korean COPD patients’ survey data. Unlike previous mapping studies that used data collected from two or more preceding randomized clinical trial studies, this study used survey data conducted at three tertiary teaching hospital. Under the Korean healthcare system, patients are not obligated to visit a general practitioner to get a referral and are free to visit any hospital. Under this circumstances, patients without serious symptoms are allowed to visit tertiary hospitals. For this reason, symptoms of COPD can vary in severity, even if the data is collected from a tertiary hospital.

The mapping model can estimate the effects of lung health on QoL, and it can be used for economic evaluations that require a quantitative utility measure. RMSE and MAE results, which serve as indicators of algorithm performances, were comparable to previous mapping studies in COPD [8–12]. To identify appropriate models, we investigated a wide range of mapping algorithms, including OLS, GLM, Tobit, beta regression, and two part models. In addition, we used the bootstrap method to generate robust estimates of RMSE and MAE in the limited sample size of 299 patients. Based on the results, OLS regression models are the recommended mapping algorithms as more complex models failed to improve results.

The recommended OLS models estimated the overall mean of EQ-5D-3 L utilities accurately. However, the recommended and the other mapping algorithms used in this study overestimated EQ-5D-3 L utilities for low utility patients (< 0.6). The predicted EQ-5D-3 L utilities have a floor effect of 0.6. The cause of this floor effect could be from the conceptual overlap and differences between CAT and EQ-5D. The three CAT items about cough (Q1), phlegm (Q2) and sleep (Q7) were little correlated with each five EQ-5D-3 L dimension, conceptually and in practice. And, the pain/discomfort dimension of EQ-5D-3 L were little correlated with each eight CAT item. These differences between CAT and EQ-5D means a limitation of predicting EQ-5D using CAT. Additional causes of the floor effect could be (i) the lack of women in the sample (Table 1) and (ii) the lack of severe patients in the sample that only 11 patients in the sample had utilities below 0.6 (Fig. 1). This overestimation for patients with severe health states has been reported in previous mapping studies; as such, this is recognized as a general problem with mapping studies [8–12]. Therefore, when using the mapping models to predict EQ-5D-3 L utilities by severity, these predictions are likely to be biased.

This study is the first mapping study of Korean patients with COPD. We conducted iterated estimation using the bootstrap method. The general procedure for model development consisted of sample splitting, training, and validating. The validation step is a weak point because the model performance ranking based on goodness-of-fit can change if the split sample changes. Therefore, selecting a model based on a single result has a probability of flawed model selection (as the model will be well-fitted to the specific validation set only, but not well-fitted generally). The iterated estimation process using the bootstrap method limits the impact of sample split and reduces the probability of poor model selection. From the bootstrap method, we ranked the mapping models based on their mean RMSEs and MAEs. These rankings were the same as rankings based on RMSE and MAE estimations using the full data set. From this finding, we questioned whether the sample split is necessary in the model development process.

It is known that EQ-5D-3 L utility is not distributed as a Gaussian distribution, for which OLS is suitable. Many previous mapping studies considered other algorithms as more suitable alternatives for the censored distribution of EQ-5D-3 L utility. However, OLS-based algorithms were recommended by most (80%) of the previous mapping studies as well as this study [15]. Because the OLS estimator minimizes the sum of squared error (which minimizes RMSE), OLS shows the lowest RMSE and would be selected as the best model when RMSE is used as a criterion. Considering other algorithms, other model selection criteria are needed, not just RMSE.

The study had some limitations, including a relatively small sample size (299 patients), inclusion of few severe status patients, and access to only one survey dataset (which could not conduct external validation). This skewed distributed sample might have introduced bias in the severe patient group estimation. Despite these limitations, the mapping algorithms in this study can be used to predict the mean level of EQ-5D-3 L utility in similar COPD patient groups.

## Conclusions

In this study, mapping algorithms were capable of predicting EQ-5D-3 L utility from the CAT of this population and comparable patient populations. The algorithms predicted overall, yet not in severe groups, mean EQ-5D-3 L utilities accurately, but these predictions in very severe groups were likely to be biased. Therefore, mapping algorithms should be used cautiously when calculated for severe disease groups.

## Additional files

Additional file 1: Supplement 1: Comparison of the models based on RMSE, MAE (full sample). Supplement 2. Comparison of the models based on the means of RMSEs, MAEs from 10,000 times iterated estimation. (R 22 kb)

Additional file 2: Mapping EQ-5D using the CAT. (R 23 kb)

## Abbreviations

CAT: COPD assessment test
CCQ: Clinical COPD questionnaire
COPD: Chronic obstructive pulmonary disease
CUA: Cost utility analysis
EQ-5D: EuroQol-5 dimension
FEV1: First second of forced expiration
FVC: Full forced vital capacity
GLM: Generalized linear model
MAE: Mean absolute error
OLS: Ordinary least squares
QALY: Quality adjusted life year
RMSE: Root mean square error
SGRQ: St. George’s Respiratory Questionnaire
